# Identifying risk factors for acute respiratory distress syndrome in critically ill patients: a retrospective study

**DOI:** 10.3389/fmed.2024.1469291

**Published:** 2024-10-02

**Authors:** Yang Zhou, Congye Li, Shuya Mei, Qiaoyi Xu, Shaojie Qin, Jinhua Feng, Jiemin Wang, Shunpeng Xing, Wei Wang, Feng Li, Quanhong Zhou, Zhengyu He, Yuan Gao, Xiaolin Zhang, Zhiyun Zhang

**Affiliations:** ^1^Department of Critical Care Medicine, Ren Ji Hospital, Shanghai Jiao Tong University School of Medicine, Shanghai, China; ^2^Department of Critical Care Medicine, Shanghai Sixth People's Hospital Affiliated to Shanghai Jiao Tong University School of Medicine, Shanghai, China; ^3^Department of Respiratory and Critical Care Medicine, Shanghai Public Health Clinical Center, Fudan University, Shanghai, China

**Keywords:** ARDS, critically ill patients, retrospective study, T lymphocyte, interleukin-6

## Abstract

**Background:**

Acute respiratory distress syndrome (ARDS) is a life-threatening condition that can develop in critically ill patients. Early identification of risk factors associated with ARDS development is essential for timely intervention and improved patient outcomes. This study aimed to investigate the potential predictors of ARDS in critically ill patients admitted to the intensive care unit (ICU).

**Methods:**

We conducted a retrospective study involving 502 critically ill patients admitted to the ICUs of three hospitals. Demographic and clinical data, including laboratory test results, were collected during their ICU stay. Multivariable logistic regression analysis was performed to identify independent risk factors associated with the development of ARDS.

**Results:**

Among the 502 critically ill patients, 104 (20.7%) patients developed ARDS during their ICU stay, with a median time to development of 5.2 days. Multivariable logistic regression analysis revealed that age (odds ratio [OR], 1.07; 95% confidence interval [CI], 1.01–1.13; *P* = 0.002), C-reactive protein (CRP) levels (OR, 1.11; 95% CI, 1.05–1.17; *P* = 0.013), T lymphocyte count (OR, 0.82; 95% CI, 0.69–0.93; *P* = 0.011), and interleukin-6 (IL-6) levels (OR, 1.17; 95% CI, 1.08–1.23; *P* = 0.003) were independently associated with the development of ARDS in critically ill patients.

**Conclusions:**

Our study identified age, CRP, T lymphocyte count, and IL-6 as independent predictors of ARDS in critically ill patients admitted to the ICU. These findings highlight the importance of monitoring these parameters in critically ill patients to identify those at high risk of developing ARDS. Early recognition and intervention based on these risk factors may improve patient outcomes in the ICU setting. Further prospective studies are warranted to validate these results and develop a reliable predictive model for ARDS in critically ill patients.

## Introduction

Acute respiratory distress syndrome (ARDS) is a severe and life-threatening condition characterized by acute onset of hypoxemia, bilateral pulmonary infiltrates, and non-cardiogenic pulmonary edema (1). It is a common complication in critically ill patients admitted to the intensive care unit (ICU) and is associated with high mortality rates, ranging from 30% to 60% (2, 3). The pathophysiology of ARDS involves a complex interplay of inflammation, endothelial dysfunction, and alveolar epithelial injury, leading to increased pulmonary vascular permeability and impaired gas exchange (4).

Early identification of patients at high risk of developing ARDS is crucial for timely intervention and improved outcomes. Several risk factors have been identified, including sepsis, pneumonia, aspiration, and trauma (5, 6). However, the predictive value of these factors varies, and there is a need for more reliable predictors to stratify patients based on their risk of developing ARDS.

Recent studies have investigated the potential role of various biomarkers in predicting ARDS development. C-reactive protein (CRP), a marker of systemic inflammation, has been shown to be elevated in patients with ARDS and may serve as a predictive marker (7, 8). Similarly, interleukin-6 (IL-6), a pro-inflammatory cytokine, has been associated with the severity and progression of ARDS (9, 10). Our study highlights the role of IL-6 and CRP in the development of ARDS, with uncontrolled inflammation being the primary cause. During inflammatory reactions, IL-6 is released in large quantities, leading to endothelial damage and increased permeability of the alveolar-capillary barrier (11). CRP has been classified as an acute-phase protein, and there is evidence that it plays a functional role in the development of ARDS (12).

Lymphopenia, particularly a decrease in T lymphocyte count, has also been observed in patients with ARDS and may reflect the severity of the underlying immune dysfunction (13, 14).

Despite these findings, the predictive value of these biomarkers in critically ill patients remains unclear, and there is a need for comprehensive studies to investigate their potential role in predicting ARDS development. Therefore, the aim of this study was to identify independent risk factors associated with ARDS development in critically ill patients admitted to the ICU and to develop a predictive model based on these factors. We hypothesized that a combination of demographic, clinical, and laboratory parameters could provide a reliable tool for early identification of patients at high risk of developing ARDS, allowing for timely intervention and improved patient outcomes.

## Methods

### Study design and population

This retrospective study was conducted in the intensive care units (ICUs) of three tertiary hospitals. Adult patients (≥18 years old) admitted to the ICUs were screened for eligibility. Patients were included if they had complete demographic, clinical, and laboratory data. Patients with pre-existing ARDS, those with incomplete data, or those who were transferred from other hospitals were excluded. The study protocol was approved by the institutional review boards of the participating hospitals, and the requirement for informed consent was waived due to the retrospective nature of the study.

### Data collection

Demographic, clinical, and laboratory data were extracted from the electronic medical records of the patients. Patient demographics, including age, sex, and BMI, were recorded at the time of ICU admission. Clinical data included comorbidities (hypertension, diabetes, chronic obstructive pulmonary disease [COPD], coronary heart disease, cerebral infarction, autoimmune disease, and history of surgery), infection sites, vital signs (body temperature, systolic blood pressure [SBP], diastolic blood pressure [DBP], and heart rate [HR]), severity of illness (assessed by Sequential Organ Failure Assessment [SOFA] score and Acute Physiology and Chronic Health Evaluation II [APACHE II] score), and use of vasopressors. Laboratory data included procalcitonin, C-reactive protein (CRP), white blood cell count, hemoglobin, platelet count, bilirubin, creatinine, lactate, brain natriuretic peptide (BNP), troponin I (TNI), uric acid, coagulation parameters (activated partial thromboplastin time [APTT], prothrombin time [PT], thrombin time [TT], international normalized ratio [INR], D-dimer, and fibrinogen), and lymphocyte subsets (CD4+ T cells, CD8+ T cells, total T cells, B cells, and natural killer [NK] cells).

### Outcome definition

The primary outcome was the development of ARDS during ICU stay, which was diagnosed according to the Berlin definition: (1) acute onset within 1 week of a known clinical insult or new or worsening respiratory symptoms; (2) bilateral opacities on chest imaging not fully explained by effusions, lobar/lung collapse, or nodules; (3) respiratory failure not fully explained by cardiac failure or fluid overload; and (4) PaO_2_/FiO_2_ ≤ 300 mmHg with positive end-expiratory pressure (PEEP) or continuous positive airway pressure (CPAP) ≥5 cmH_2_O.

### Statistical analysis

Continuous variables were presented as median (interquartile range [IQR]) and compared using the Mann-Whitney U test. Categorical variables were presented as number (percentage) and compared using the chi-square test or Fisher's exact test, as appropriate. Patients were randomly divided into a training cohort (80%) and a validation cohort (20%). In the training cohort, univariate logistic regression analysis was performed to identify potential risk factors for ARDS development. Variables with *P* < 0.1 in the univariate analysis were included in the multivariate logistic regression analysis to identify independent risk factors, after single-factor analysis, factors with a *p*-value < 0.1 were included in the multivariate analysis, and outcomes with a *p*-value < 0.05 were considered to be associated with the outcome. A predictive model was constructed based on the independent risk factors identified in the multivariate analysis. The performance of the predictive model was evaluated using receiver operating characteristic (ROC) curve analysis in the training cohort, validation cohort, and entire cohort. The area under the ROC curve (AUC) was calculated to assess the discriminatory ability of the model. All statistical analyses were performed using R software (version 4.0.3), and a two-tailed *P* < 0.05 was considered statistically significant.

## Results

### Baseline characteristics

**Figure 1:**
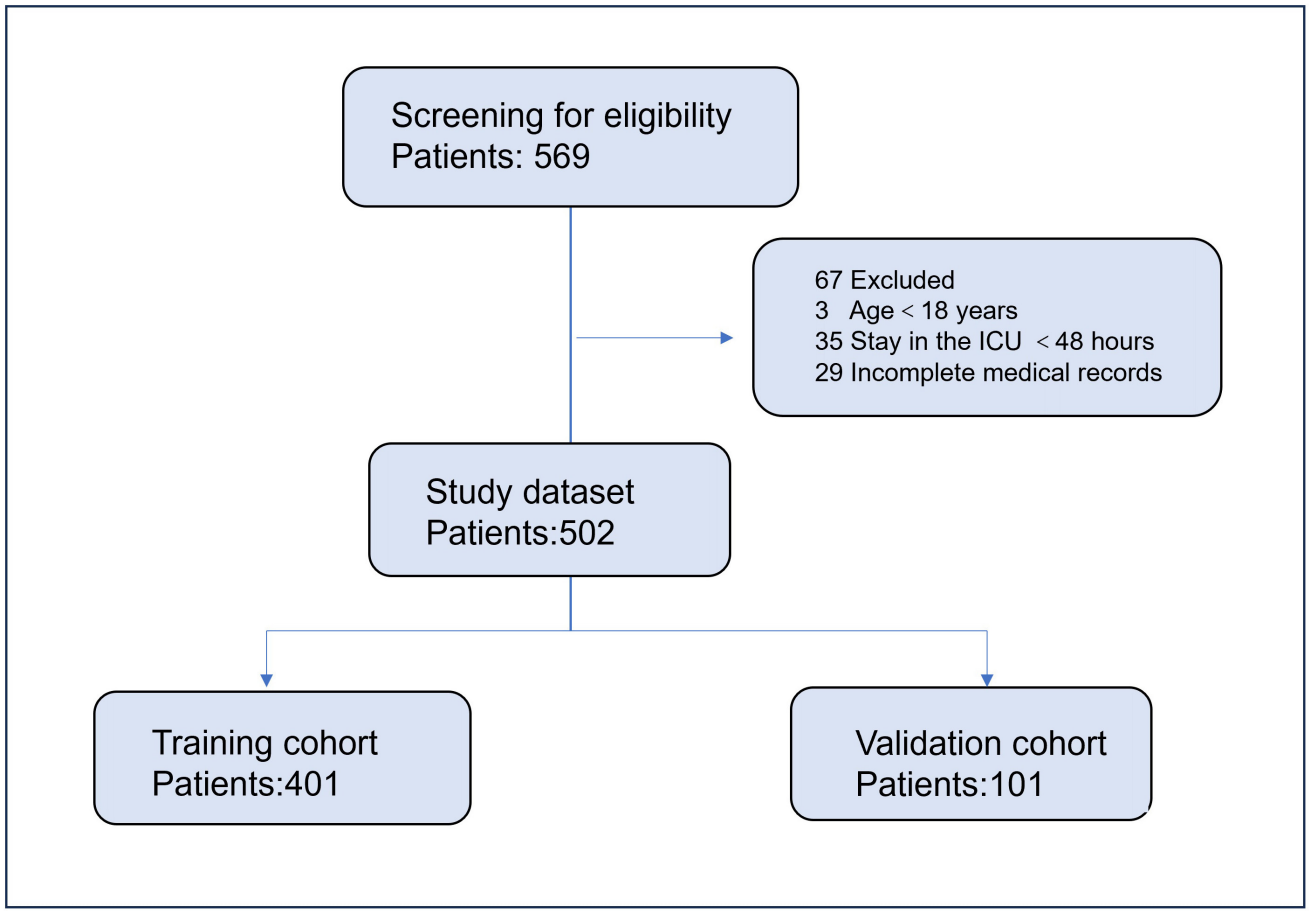
Flowchart of patient selection process.

A total of 502 critically ill patients were included in the study, with 401 patients in the training cohort and 101 patients in the validation cohort (Figure 1). The median age was 63 years (IQR, 48–72 years), and 54.9% of the patients were male. The most common comorbidities were hypertension (30.8%), autoimmune disease (33.6%), and diabetes (22.0%). The respiratory tract was the most common site of infection (68.5%). The median SOFA score was 6 (IQR, 3–10), and the median APACHE II score was 16 (IQR, 11–22). Vasopressors were used in 42.7% of the patients. There were no significant differences in baseline characteristics between the training and validation cohorts (Table 1).

**Table 1:** Baseline characteristics of included patients.

**Characteristics**	**Total (*n =* 502)**	**Train cohort (*n =* 401)**	**Validation cohort (*n =* 101)**	***P* value**
**Demographic variables**				
Age (years)	63 (48,72)	64 (47,74)	62 (49,72)	0.885
Gender, *n* (%)				0.779
Male	54.90%	54.10%	56.70%	
Female	45.10%	45.90%	43.30%	
BMI (kg/m2)	26.1 (23.7, 31.9)	26.0 (23.5, 31.5)	26.3 (23.8, 32.0)	0.811
**Comorbidities**				
Hypertension, *n* (%)	30.80%	28.60%	35.60%	0.293
Diabetes, *n* (%)	22.00%	20.90%	24.40%	0.607
COPD, *n* (%)	5.60%	6.60%	3.30%	0.607
Coronary heart disease, *n* (%)	5.60%	9.70%	8.90%	1
Cerebral infarction, *n* (%)	11.90%	10.70%	14.40%	0.479
Autoimmune disease, *n* (%)	33.60%	36.20%	27.80%	0.204
History of surgery, *n* (%)	15.00%	14.80%	15.60%	1
**Infection sites**				
Respiratory tract, *n* (%)	68.50%	68.40%	68.90%	1
Urinary tract, *n* (%)	13.30%	11.20%	17.80%	0.184
Gastrointestinal tract, *n* (%)	6.70%	7.10%	6.70%	1
Liver, *n* (%)	11.90%	10.70%	14.40%	0.479
Skin and soft tissues, *n* (%)	4.90%	5.60%	3.30%	0.593
CNS, *n* (%)	2.50%	3.10%	1.10%	0.562
Unknown, *n* (%)	5.20%	5.60%	4.40%	0.9
**Vital signs**				
Body temperature (°C)	38.2 (37.0 to 39.2)	38.2 (37.0 to 39.0)	38.30 (37.10 to 39.40)	0.117
SBP (mmHg)	119 (104 to 135)	118 (103 to 131)	122 (104 to 140)	0.199
DBP (mmHg)	70 (61 to 80)	70 (61 to 78)	74 (61 to 82)	0.131
HR (/min)	96 (91,102)	96 (92,103)	95 (90,104)	0.677
**Severity of disease**				
SOFA	6 (3 to 10)	6 (3 to 10)	6 (3 to 10)	0.601
APACHE II	16 (11 to 22)	16 (12 to 22)	17 (10 to 22)	0.862
Use of vasopressors, *n* (%)	122 (42.7%)	83 (42.3%)	39 (43.3%)	0.978
**Laboratory test**				
Procalcitonin (ng/ml)	2.34 (0.38 to 9.42)	2.28 (0.37 to 7.84)	2.60 (4010.47 to 20.40)	0.203
C-reactive protein (mg/L)	88.28 (25.82 to 192.53)	79.29 (24.52 to 176.92)	105.77 (32.32 to 200.00)	0.171
White blood cells (10^∧^9/L)	10.25 (7.01 to 13.73)	10.06 (7.09 to 13.52)	10.35 (6.75 to 13.94)	0.751
Hemoglobin (g/L)	119.5 (100.0 to 134.0)	120.00 (99.0 to 135.5)	119.00 (102.0 to 134.0)	0.693
Platelet (10^∧^9/L)	157.50 (96.00 to 217.00)	162.50 (100.50 to 224.50)	142.00 (93.00 to 204.00)	0.153
Bilirubin (umol/L)	17.85 (12.00 to 31.00)	17.65 (11.70 to 30.00)	19.00 (12.00 to 31.70)	0.823
Creatinine (umol/L)	98.50 (71.00 to 120.00)	95.50 (68.50 to 119.50)	100.00 (71.00 to 122.00)	0.482
Lactate (mmol/L)	2.33 (1.60 to 3.48)	2.40 (1.63 to 3.40)	2.20 (1.52 to 4.10)	0.961
BNP (pg/ml)	124.50 (43.10 to 420.00)	124.50 (43.45 to 450.00)	124.50 (35.70 to 402.00)	0.875
TNI (ng/ml)	0.04 (0.04 to 0.09)	0.04 (0.04 to 0.07)	0.04 (0.04 to 0.21)	0.2
Uric acid (umol/L)	338.00 (246.00 to 429.00)	343.00 (246.00 to 445.50)	324.00 (246.00 to 417.00)	0.318
APTT (sec)	32.60 (27.60 to 41.00)	32.55 (27.60 to 39.50)	33.50 (27.40 to 45.80)	0.253
PT (sec)	12.85 (11.70 to 14.60)	12.80 (11.65 to 14.40)	12.95 (11.80 to 15.30)	0.382
TT (sec)	18.40 (17.10 to 20.40)	18.45 (17.25 to 20.55)	18.10 (17.10 to 19.90)	0.104
INR	1.09 (0.99 to 1.25)	1.08 (0.98 to 1.23)	1.10 (1.00 to 1.31)	0.351
D-D (ug/ml)	2.88 (1.23 to 6.52)	2.27 (1.10 to 5.00)	2.46 (1.26 to 4.38)	0.204
Fibrinogen (g/L)	4.35 (2.54 to 5.78)	4.37 (2.54 to 5.72)	4.33 (2.54 to 5.78)	0.763
CD4+ T cells (cells/uL)	260.50 (180.00 to 466.00)	265.5 (172.5 to 468.0)	257.0 (186.0 to 402.0)	0.683
CD8+ T cells (cells/uL)	206.50 (122.00 to 285.00)	215.0 (127.0 to 289.0)	215.0 (127.0 to 289.0)	0.384
T cells (cells/uL)	480.00 (291.00 to 763.00)	487.00 (297.50 to 804.00)	457.50 (272.00 to 708.00)	0.509
B cells (cells/uL)	99.00 (46.00 to 225.00)	96.00 (45.50 to 223.50)	100.50 (51.00 to 242.00)	0.577
NK cells (cells/uL)	124.00 (65.00 to 204.00)	125.00 (67.50 to 210.00)	116.50 (64.00 to 202.00)	0.524

BMI, Body mass index; COPD, Chronic Obstructive pulmonary disease; CNS, Central Nervous System; SBP, Systolic Blood Pressure; DBP, Diastolic Blood Pressure; HR, Heartrate; SOFA, Sequential Organ Failure Assessment; APACHE II, Acute Physiology And Chronic Health Evaluation II; BNP, Brain Natriuretic Peptide; TNI, Troponin I; APTT, Activated Partial thromboplastin Time; PT, Prothrombin Time; TT, Thrombin Time; INR, International Normalized Ratio; D-D, D-Dimer; NK, Natural Killer.

### Univariate and multivariate analyses

**Figure 2:**
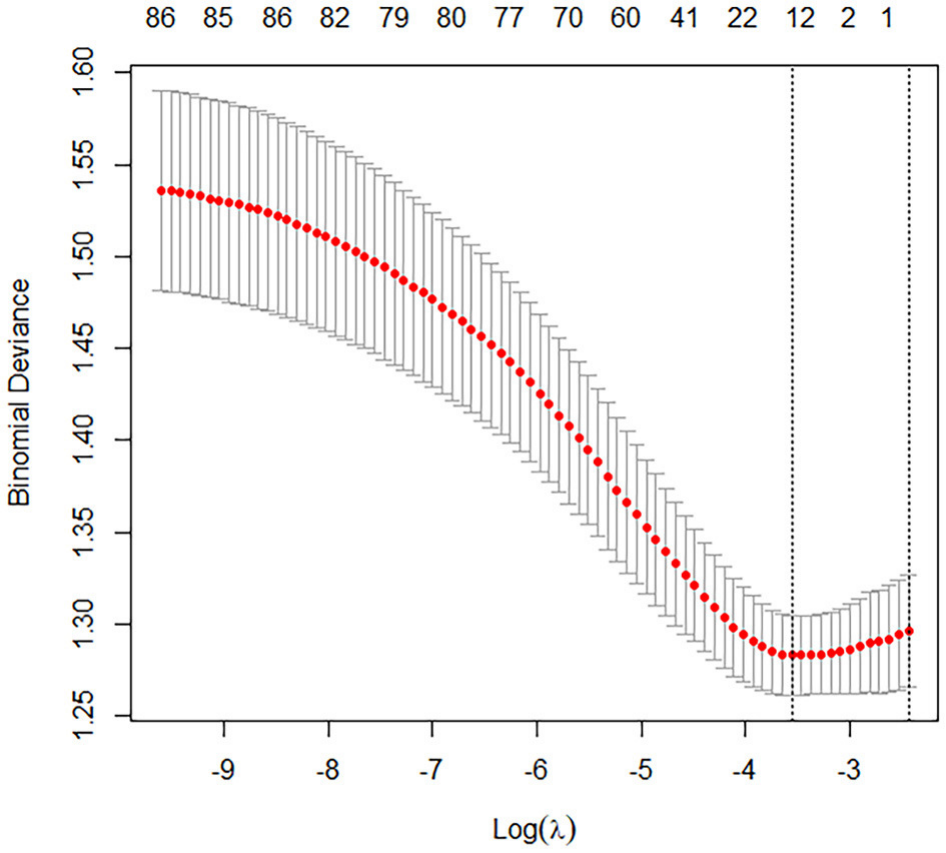
Binomial deviance plot for model tuning with cross-validation.

**Figure 3:**
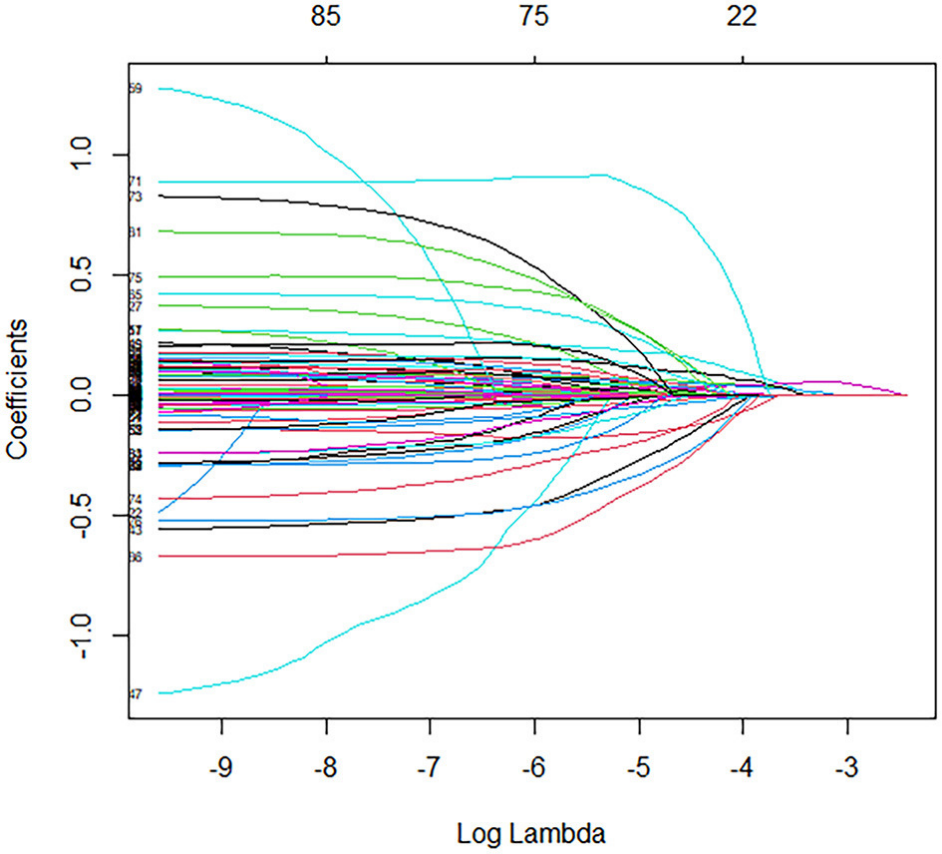
Coefficient paths for LASSO regression.

In the training cohort, univariate logistic regression analysis identified 12 variables with *P* < 0.1, including age, BMI, SOFA score, APACHE II score, use of vasopressors, procalcitonin, CRP, lactate, TNI, APTT, D-dimer, and IL-6 (Figures 2, 3). These variables were included in the multivariate logistic regression analysis, which revealed four independent risk factors for ARDS development: age (odds ratio [OR], 1.07; 95% confidence interval [CI], 1.01–1.13; *P* = 0.002), C-reactive protein (CRP) levels (OR, 1.11; 95% CI, 1.05–1.17; *P* = 0.013), T lymphocyte count (OR, 0.82; 95% CI, 0.69–0.93; *P* = 0.011), and interleukin-6 (IL-6) levels (OR, 1.17; 95% CI, 1.08–1.23; *P* = 0.003) (Table 2).

**Table 2:** Multivariate logistic regression analysis of risk factors of ARDS based on selected variables in the training cohort.

**Variable**	**OR (95% CI)**	***P* value**
Age	1.07 (1.01, 1.13)	0.002
CRP	1.11 (1.05, 1.17)	0.013
T lymphocyte	0.82 (0.69, 0.93)	0.011
IL-6	1.17 (1.08, 1.23)	0.003

OR, odds ratio; CI, confidence interval.

### Predictive model and performance

**Figure 4:**
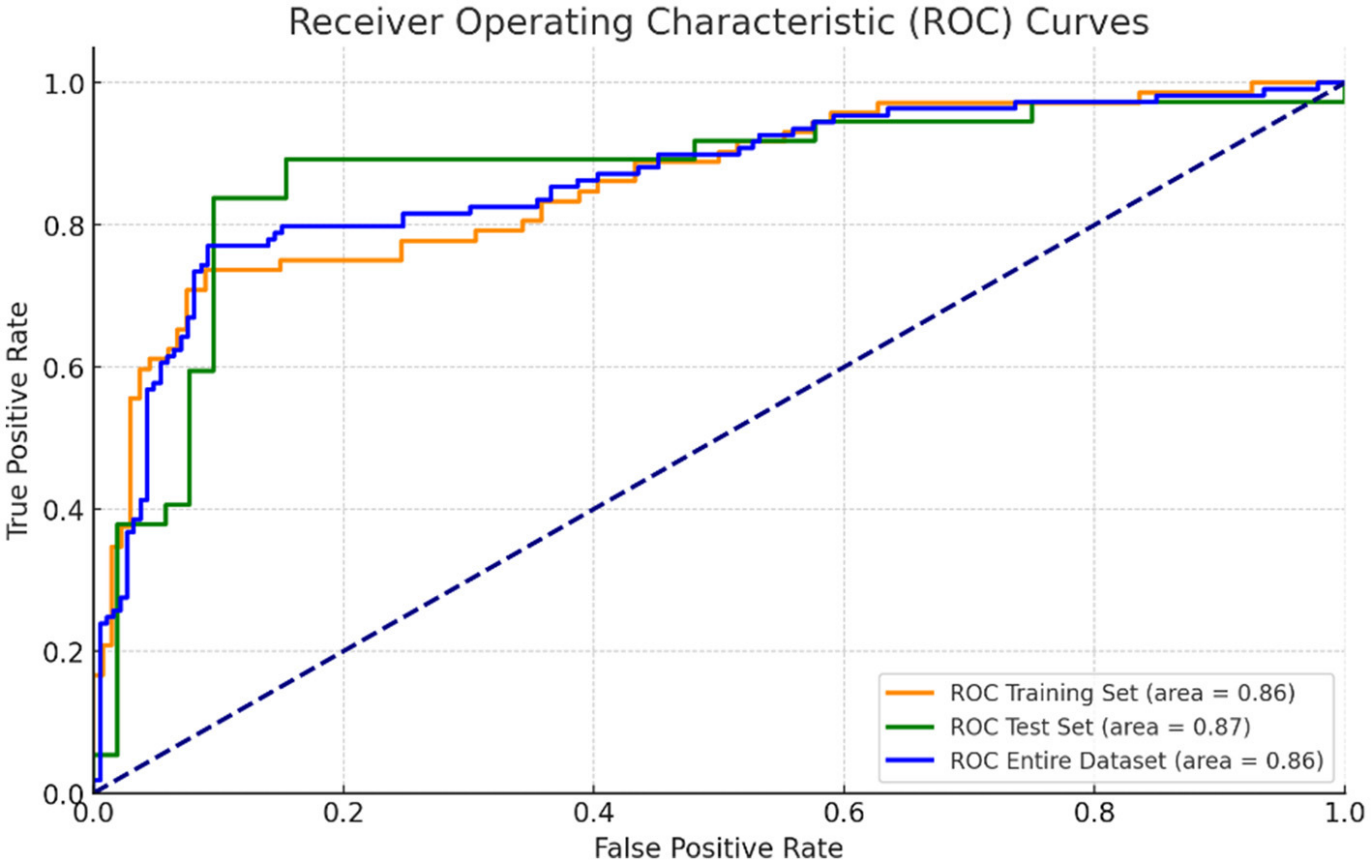
ROC curves for predictive model performance in training, validation, and entire dataset.

**Figure 5:**
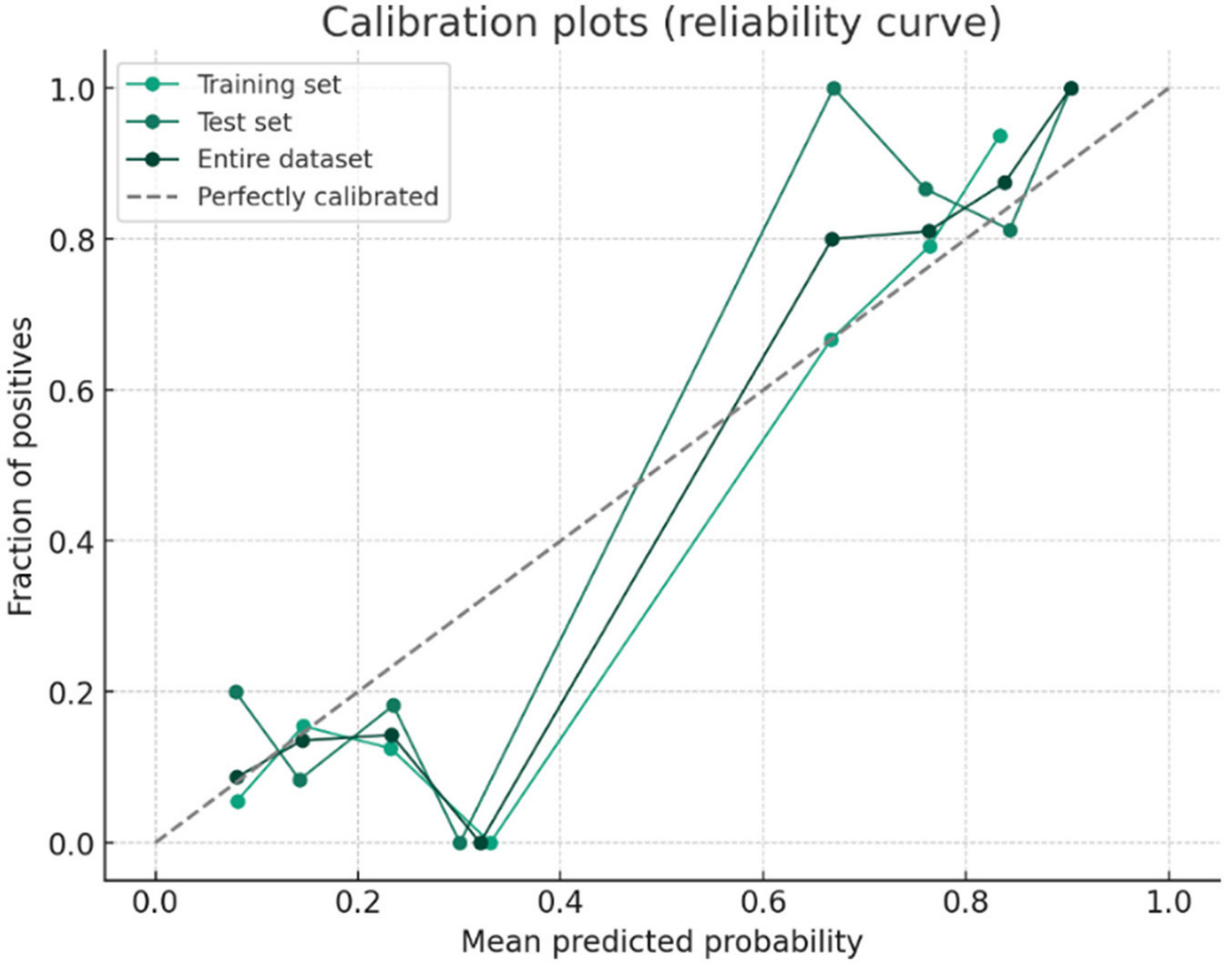
Calibration plot of the predictive model.

A predictive model for ARDS development was constructed based on the four independent risk factors identified in the multivariate analysis. The ROC curves of the model in the training cohort, validation cohort, and entire cohort are shown in Figure 4. The AUC was 0.86 (95% CI, 0.83–0.91) in the test cohort, 0.86 (95% CI, 0.78–0.94) in the validation cohort, and 0.86 (95% CI, 0.83–0.90) in the entire cohort, indicating excellent discriminatory ability of the model in identifying patients at high risk of developing ARDS (Figures 4, 5).

## Discussion

In this retrospective study, we identified age, CRP, T lymphocyte count, and IL-6 as independent predictors of ARDS development in critically ill patients admitted to the ICU. These findings highlight the importance of considering both demographic and laboratory parameters in assessing the risk of ARDS in this patient population.

Age has been consistently identified as a risk factor for ARDS in previous studies (15, 16). Our study further confirms this association, with older patients being more likely to develop ARDS during their ICU stay. This may be attributed to the age-related changes in the immune system, decreased lung compliance, and increased susceptibility to infections and inflammatory conditions (17). Clinicians should be aware of the increased risk of ARDS in elderly patients and closely monitor them for early signs of respiratory distress.

CRP, a marker of systemic inflammation, was found to be an independent predictor of ARDS in our study. This is consistent with previous findings suggesting that elevated CRP levels are associated with the development and progression of ARDS (7, 8). CRP is a readily available biomarker that can be easily measured in clinical settings, making it a valuable tool for identifying patients at high risk of ARDS. Monitoring CRP levels in critically ill patients may help guide early interventions and optimize patient management.

Our study also identified T lymphocyte count as an independent predictor of ARDS. Lymphopenia, particularly a decrease in T lymphocytes, has been observed in patients with ARDS and is thought to reflect the severity of the underlying immune dysfunction (13, 14). The depletion of T lymphocytes may impair the body's ability to mount an effective immune response against pathogens and regulate the inflammatory process, contributing to the development of ARDS. Monitoring T lymphocyte count in critically ill patients may provide valuable insights into their immune status and help identify those at high risk of ARDS.

IL-6, a pro-inflammatory cytokine, was found to be independently associated with ARDS development in our study. This finding is in line with previous studies suggesting that elevated IL-6 levels are associated with the severity and progression of ARDS (9, 10). IL-6 plays a central role in the inflammatory cascade and may contribute to the pathogenesis of ARDS by promoting endothelial dysfunction, alveolar epithelial injury, and the recruitment of inflammatory cells (18). Measuring IL-6 levels in critically ill patients may help identify those with a heightened inflammatory response and increased risk of ARDS.

One of the strengths of our study is the large sample size, which allowed us to investigate multiple potential predictors of ARDS in critically ill patients. Additionally, we used a rigorous statistical approach, including multivariable logistic regression analysis, to identify independent risk factors while controlling for potential confounders. This approach provides a more comprehensive understanding of the complex interplay between various factors contributing to ARDS development.

However, our study has several limitations. First, this study was retrospective, which inevitably led to heterogeneity among patients' characteristics. However, by comparing the baseline features between the training set and validation set, we were able to mitigate this limitation. Second, we did not have data on the specific causes of ARDS in our patient population, which may have influenced the predictive value of the identified risk factors. Finally, although our study was conducted in a multicenter setting, the sample size limitations may restrict the generalizability of our findings to other patient populations.

In conclusion, our study identified age, CRP, T lymphocyte count, and IL-6 as independent predictors of ARDS development in critically ill patients admitted to the ICU. These findings highlight the importance of considering both demographic and laboratory parameters in assessing the risk of ARDS and may help guide early interventions and optimize patient management. Further prospective studies are warranted to validate these results and develop a reliable predictive model for ARDS in critically ill patients.

## Data Availability

The original contributions presented in the study are included in the article/supplementary material, further inquiries can be directed to the corresponding authors.
